# Enhanced management strategy of synchronous percutaneous biopsy and microwave ablation in patients with lung ground-glass opacities undergoing antithrombotic treatment: a clinical perspective on our experience

**DOI:** 10.3389/fonc.2025.1554365

**Published:** 2025-07-23

**Authors:** Nan Wang, Guoqiang Chen, Bingjie Jin, Wenjing Lu, Jie Xu, Jingwen Xu, Guoliang Xue, Xin Ye

**Affiliations:** ^1^ Department of First Clinical Medical College, Shandong University of Traditional Chinese Medicine, Shandong Provincial Qianfoshan Hospital, Jinan, Shandong, China; ^2^ Department of Oncology, The First Affiliated Hospital of Shandong First Medical University and Shandong Provincial Qianfoshan Hospital, Shandong Lung Cancer Institute, Jinan, China; ^3^ Department of Gastroenterology, Shandong Provincial Hospital Affiliated to Shandong First Medical University, Jinan, China; ^4^ Department of Radiology, People’s Hospital of Guangrao County, Dongying, Shandong, China; ^5^ Shandong Medicine and Health Key Laboratory of Cardiac Electrophysiology and Arrhythmia, Department of Cardiology, The First Affiliated Hospital of Shandong First Medical University & Shandong Provincial Qianfoshan Hospital, Jinan, Shandong, China

**Keywords:** microwave ablation, biopsy, ground-glass opacities, antithrombotic therapy, complication

## Abstract

**Objective:**

This retrospective study was conducted to delineate our experience in managing perioperative antithrombotic agents in patients receiving antithrombotic therapy underwent percutaneous biopsy and microwave ablation (B+MWA) for lung ground-glass opacities (GGOs).

**Methods:**

The study comprised 67 patients with GGOs who receiving antithrombotic therapy underwent B+MWA sessions from January 1, 2020, to May 31, 2022. During the perioperative period, patients who received rivaroxaban as a bridging drug were assigned to Group A, and who interrupted the antithrombotic therapy were assigned to Group B. Information about the technical success rate, positive biopsy rate, local control rates, and major bleeding and thrombotic complications were collected and analyzed.

**Results:**

Group A comprised 36 patients (19 males; mean age, 67.97 ± 8.49 years), while Group B comprised 31 patients (12 males; mean age, 65.48 ± 4.32 years). The technical success rate was 100%. The positive biopsy rates were 94.44% and 96.77%, respectively. In group A and B, the overall local control rates at 6, 18, and 24 months were 100.0% vs. 100.0%, 94.44% (34/36) vs. 96.77% (30/31), and 86.11% (31/36) vs. 87.10% (27/31), with no significant difference between the two groups (p = 0.2156). During the perioperative period, a single case of lower extremity venous thrombosis was identified in Group A, while three cases of lower extremity venous thrombosis, one case of new-onset cerebral infarction, and one case of new-onset pulmonary embolism were identified in Group B, with no statistically significant difference in the overall incidence of bleeding and thrombotic complications between the two groups.

**Conclusions:**

Compared with direct interruption of antithrombotic therapy, the use of rivaroxaban in the perioperative period of B+MWA in patients with GGOs who are receiving antithrombotic therapy can reduce the incidence of severe thrombotic complications without increasing the risk of bleeding, with a satisfactory effectiveness.

## Highlights

The significant finding of the study is that bridging oral anticoagulant therapy along with synchronous CT-guided percutaneous biopsy and microwave ablation should be considered as an alternative treatment for lung ground-glass opacities in patients receiving antithrombotic therapy. This study adds the research data of microwave ablation for patients receiving antithrombotic therapy.

## Introduction

Ground-glass opacity (GGO) is imaging manifestations on computed tomography (CT) that appear as obscure shadows covering the underlying bronchial structure and vascular structure of the lung. GGO is a nonspecific imaging parameter for early-stage pulmonary cancer (1, 2). Highly suspicious lung ground-glass opacities (HSML-GGOs) are generally defined as nonsolid pulmonary nodules >6mm or with increasing solid components that persist during a 6–9 months follow-up, which are difficult to detect on chest X-ray. However, with the wide application of computed tomography (CT) in recent decades, HSML-GGOs have attracted increasing attention (3, 4). HSML-GGOs usually have irregular margins, complex density, and spiculate or lobular outline (5). These imaging features make it difficult to distinguish malignant GGOs from some benign nodules, such as tuberculoma or inflammatory pseudotumor (6, 7). Moreover, due to the biological characteristics of GGOs, traditional imaging examinations such as enhanced CT, magnetic resonance imaging, Positron Emission Tomography-CT, etc., cannot well distinguish benign and malignant GGOs (5, 8). Currently, while the management of HSML-GGOs remains a topic of contention and is the subject of ongoing research, long-term follow-up data indicate that clinical guidelines recommend a tailored approach. For certain patients, this involves a period of surveillance, whereas others may require tissue sampling, including resection, based on identified risk factors (9, 10). The precise diagnosis of HSML-GGOs depends on pathological examination, which was obtained by surgical resection and percutaneous pulmonary biopsy (11–13). Percutaneous pulmonary biopsy technique has seen significant advancement, yet parenchymal hemorrhage persists as an inescapable complication (14, 15). Unlike solid nodules, the occurrence of pulmonary hemorrhage tends to have a greater impact on the visualization and localization of GGOs on CT scans. This interference can lead to unsuccessful procedure outcomes or an elevated false-negative rate in pathology results, a concern that is particularly acute for patients undergoing antithrombotic therapy. However, for patients necessitating antithrombotic treatment, prematurely discontinuing antithrombotic therapy can precipitate life-threatening thrombotic complications, particularly in elderly patients with malignant tumors who are more likely to develop thrombotic complications because of the hypercoagulable state (16, 17). Consequently, judicious management of anticoagulant medications during the period of B+MWA for GGOs is of paramount importance (18, 19). A strategy involving rivaroxaban as a bridging antithrombotic agent has been extensively investigated in clinical practice (20, 21). The objective of this study is to assess the safety and efficacy of rivaroxaban bridging in patients with GGOs underwent CT-guided percutaneous B+MWA procedure. This study will provide fresh evidence to inform the use of rivaroxaban in patients undergoing percutaneous pulmonary biopsy while on antithrombotic therapy, thereby guiding clinical decision-making.

## Materials and methods

### Patients

**Figure 1:**
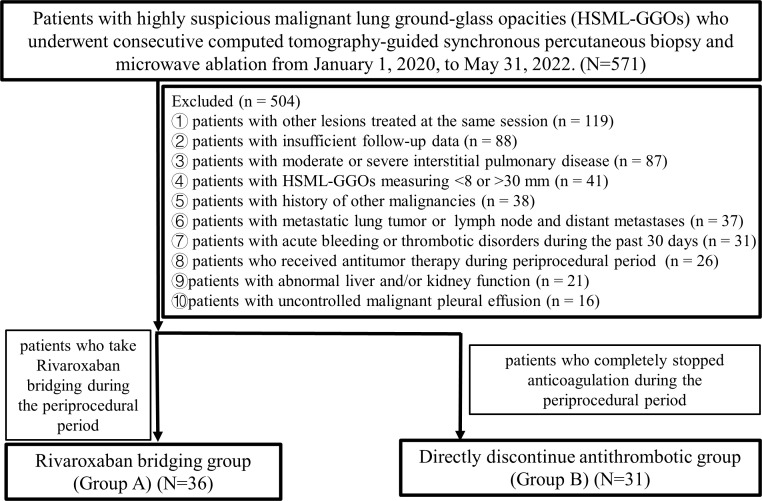
Flowchart of the patient selection criteria.

This study consisted of 486 patients on antithrombotic therapy with GGOs who underwent 571 consecutive B+MWA treatments from January 1, 2020, to May 31, 2022. These patients were unsuitable candidates for surgery or radiotherapy. The inclusion criteria were as follows: 1) patients with ground-glass nodule diagnosed as HSML-GGOs; 2) those with GGOs measuring 8–30 mm; and 3) those with an Eastern Cooperative Oncology Group performance status (ECOG PS) score of ≤ 2. The exclusion criteria were as follows: 1) patients with other lesions treated at the same session; 2) those with metastatic lung tumor or primary lung cancer with lymph node and distant metastases; 3) those with abnormal liver and/or kidney function; 4) those with acute bleeding or thrombotic disorders during the past 30 days; 5) those with moderate or severe interstitial pulmonary disease; 6) those with uncontrolled malignant pleural effusion; 7) those who received other antitumor therapy during the peri-procedural period; 8) those with history of other malignancies; and 9) those who were lost to follow-up. In total, 67 patients (comprising 31 males and 36 females; with a mean age of 66.82 ± 7.62 years, ranging from 50 to 79 years) were enrolled in the study. The process of patient selection is delineated in **Figure 1**. Patients who received rivaroxaban bridging during their hospitalization for B+MWA treatment were allocated to Group A, while those who ceased antithrombotic therapy directly during their hospitalization were placed in Group B. Prior to treatment, all patients underwent a multidisciplinary consultation involving specialists from thoracic surgery, oncology, respiratory diseases, radiotherapy, interventional radiology, imaging, and pathology. The treatment plan was subsequently formulated in accordance with expert consensus (22).

This study complied with the standards of the Declaration of Helsinki and obtained approval from the Institutional Ethics Committee of Shandong Provincial Qianfoshan Hospital, Jinan, Shandong, China. The Institutional Review Board waived the requirement for informed consent since this was a retrospective study.

### Synchronous percutaneous biopsy and microwave ablation

The procedure of CT-guided B+MWA for patients with pulmonary nodules has been extensively documented in previous studies (23, 24). The approach here is to place the ablation antenna in a satisfactory position and then place the biopsy coaxial needle and the biopsy needle according to the preoperative plan. Ablation is strategically timed to facilitate microvascular hemostasis and thrombosis within the ablation area, thereby mitigating the risk of bleeding that may arise during the subsequent biopsy. Should the biopsy inadvertently result in excessive bleeding that compromises the visibility of the GGOs on CT imaging, the accuracy of the antenna’s position is reassessed based on its relative location to the preoperative reference markers, and ablation is then carried out accordingly. Specifically, it is divided into the following steps: 1). After local anesthesia with 5–10 mL of 2% lidocaine, a 19-G (Gauge) antenna was inserted, followed by a coaxial guiding needle (17 G, GMT Medical) along the designated path or the ablation antenna; 2). Ablation was performed for 1–2 min at 30–50 W power. CT was performed again to observe changes at the ablation site. The position of the needle tip was confirmed before the 18-G biopsy needle was inserted along the coaxial catheter, and samples were collected. If severe pulmonary bleeding was observed, ablation with a power of 30–50 W was applied for 1–2 min to promote hemostasis; 3). After performing the B+MWA, as described previously, optimal circumferential ablated margins of ≥5 mm for GGO margins were achieved; 4). The needle tract was ablated to promote hemostasis and reduce metastasis. Vessels with a diameter > 2 mm distributed along the ablation antenna path were ablated for 1–2 min at the intersection of the path and the vessel to reduce bleeding. It was not advisable to have vessels >3 mm in diameter within the preprocedural designed puncture path.

All participants were advised to bed overnight. The important observations included: 1) abnormal hypotension and tachycardia (systolic pressure < 90 mmHg, heart rate >100 beats/min); 2) unusual hemoptysis or any other manifestations or symptoms suggestive of bleeding, such as severe chest pain, dyspnea, or vertigo; 3) In such instances, immediate hemoglobin level assessment and chest CT scan were conducted, while electrocardiogram and blood pressure monitoring continued. Appropriate fluid resuscitation and hemostatic measures were initiated, and blood transfusion was considered if hemodynamic instability persisted; 4) A routine postoperative chest CT scan was scheduled 24 hours post-surgery. If the bleeding range did not diminish within 24 hours postoperatively, a CT scan was repeated every three days postoperatively until a trend of hemorrhage reduction was evident.

### Management of anticoagulants in the perioperative period

The perioperative period was defined as spanning from 5 days prior to the B+MWA procedure to 1-month post-procedure. Within this timeframe, we exclusively monitored for procedure-related bleeding and thrombotic complications. All patients discontinued all antithrombotic medications 5 days prior to procedure. Patients in group A were administered rivaroxaban (10 mg once a day) bridging, whereas patients in group B did not receive any anticoagulants. The original antithrombotic therapy could be reinitiated in patients who met the following criteria 3 days post-procedure: 1) A chest CT scan performed 24 hours or 3 days post-procedure demonstrated a reduction in the bleeding range; 2) The patient did not present with hemoptysis; 3) The patient exhibited no other new bleeding complications, such as epistaxis, gingival bleeding, visceral bleeding, or central bleeding. Patients who restarted antithrombotic therapy were to be monitored closely throughout the perioperative period. In the event of bleeding complications, immediate further examination and treatment would be pursued to avert fatal bleeding. Should thrombotic complications arise during the perioperative period, consultations with relevant departments would be solicited, and appropriate medication adjustments or treatments would be implemented.

### Response criteria and follow-up

Technical success was defined as completion of MWA with sample collection as planned before procedure. Positive biopsy rate was defined as diagnostic pathological findings, including atypical adenomatoid hyperplasia (AAH), adenocarcinoma *in situ* (AIS), inflammatory tissue, and malignant tissue. Chest enhanced CT was performed at 1-, 3-, 6-, 12-, 18- and 24-months after procedure. Complete ablation rate was used to describe lesion control rate. The severity of complications was evaluated according to the National Cancer Institute Common Terminology Criteria for Adverse Events (CTCAE; version 5.0), and grade ≥3 complications were defined as major complications (25).

### Statistical analysis

Statistical analyses were performed using SPSS version 20.0. Categorical variables were compared using Fisher’s exact test, whereas continuous variables were compared using the unpaired Student’s t-test (parametric) or Mann-Whitney U test (nonparametric). P values <0.05 in a two-tailed test were considered statistically significant. All data used in this study were recorded for reference purposes.

## Results

**Table 1** shows the baseline characteristics of the patients. There were 36 patients (19 men; mean age, 67.97 ± 8.49 years) in Group A and 31 patients (12 men; mean age, 65.48 ± 4.32 years) in Group B. There were no significant differences between the two groups in terms of the age, gender, lesion’s location, lesion’s diameter, distance of lesion to great vessels, lesion’s diagnosis, smoking rate, previous history of thrombosis and bleeding, history of vascular stent implantation, and preprocedural coagulation function. The preprocedural coagulation function was defined as the coagulation profile assessed within 5 days before the procedure. **Table 2** shows the procedural characteristics. There are no significant differences in patient position, puncture path length, puncture direction, cases of puncture though vessel’s diameter > 2mm, mean maximum ablation power, mean ablation application duration, mean total procedure time, and hospitalization days between the two groups.

**Table 1:** Baseline characteristics of patients.

Characteristics	Group A (n = 36)	Group B (n = 31)	P-value
Age (years)	67.97 ± 8.49	65.48 ± 4.32	0.1844
Male gender, n (%)	19 (52.78)	12 (38.71)	0.6772
Lesion location, n (%)			0.7699
Central location*	9 (25.00)	6 (19.35)	
Peripheral location	27 (75.00)	25 (80.65)	
Maximum lesion diameter (mm)	17.94 ± 6.45	18.74 ± 6.51	0.6169
Distance from great vessels (>3 mm) , n (%)			0.3197
≤10 mm	7 (19.44)	3 (9.68)	
>10 mm	29 (80.56)	28 (90.32)	
Diagnosis of lesions, n (%)			0.2276
Malignancy	26 (72.22)	24 (74.22)	
Not malignancy	10 (27.78)	7 (25.78)	
Smoking, n (%)			0.5673
Yes	10 (27.78)	6 (19.35)	
No	26 (72.22)	25 (80.65)	
Previous history of thrombosis, n (%)			0.5916
Yes	9 (25.00)	10 (32.26)	
No	27 (75.00)	21 (67.74)	
Previous history of bleeding, n (%)			0.3221
Yes	4 (11.11)	7 (22.58)	
No	32 (88.89)	24 (77.42)	
History of vascular stent implantation, n (%)			0.7946
Yes	24 (66.67)	22 (70.97)	
No	12 (33.33)	9 (29.03)	
Antithrombotic drug regimens, n (%)			>0.9999
Single agent	30 (83.33)	26 (83.87)	
Aspirin	12 (33.3)	11 (35.5)	
Clopidogrel	8 (22.2)	6 (19.4)	
Warfarin	4 (11.1)	3 (9.7)	
Rivaroxaban	3 (8.3)	3 (9.7)	
Apixaban	2 (5.6)	2 (6.5)	
Dabigatran	1 (2.8)	1 (3.2)	
Combination medication	6 (16.67)	5 (16.13)	
Aspirin + Clopidogrel	4 (11.1)	3 (9.7)	
Aspirin + Rivaroxaban	1 (2.8)	1 (3.2)	
Clopidogrel + Warfarin	1 (2.8)	1 (3.2)	
Preprocedural coagulation function			
Prothrombin time (s)	11.00 ± 0.79	10.84 ± 0.57	0.3621
International normalized ratio	1.16 ± 0.41	1.05 ± 0.33	0.1351
D-dimer (μg/L)	0.39 ± 0.27	0.31 ± 0.18	0.1707

Quantitative variables are expressed as means ± standard deviation.

*Central location is defined as lesions touching the “zone of the proximal bronchial tree” which is a 20mm radius around the main tracheo-bronchial tree.

**Table 2:** The procedural characteristics.

Characteristics	Group A (n = 36)	Group B (n = 31)	P-value
Patient position, n (%)			>0.9999
Supine	21 (58.33)	19 (61.29)	
Prone	15 (41.67)	12 (38.71)	
Puncture path length (mm)	95.95 ± 14.59	93.61 ± 13.09	0.4943
Puncture direction, n (%)			>0.9999
Vertical to pleura*	27 (75.00)	23 (74.19)	
Parallel to pleura**	9 (25.00)	8 (25.81)	
Puncture though vessels > 2mm, n (%)			0.0964
Yes	6 (16.67)	11 (35.48)	
No	30 (83.33)	20 (64.52)	
Mean MAX ablation power (w)	38.61 ± 5.43	40.00 ± 3.65	0.2313
Mean ablation application time (s)	410.83 ± 141.45	354.77 ± 119.28	0.087
Mean total procedure time (min)	50.94 ± 16.21	51.48 ± 15.80	0.8911
Hospitalization (range), days	5.35 ± 3.04 (4–13)	6.79 ± 4.55 (5–13)	0.5372

Quantitative variables are expressed as means ± standard deviation.

*Vertical to pleural puncture was defined as an Angle greater than 30°between the antenna and the pleural tangent line of the puncture point.

**Parallel to pleural puncture was defined as an Angle less than 30°between the antenna and the pleural tangent line of the puncture point.

### Efficacy

**Table 3** shows the treatment efficacy of two groups. The mean follow-up time was 22.4 ± 7.9 months (range, 15.5–39.8 months). The technical success rate was 100%. In group A and group B, 8.33% (3/36) and 12.90% (4/31) of the lesions were pathologically diagnosed as alveolar tissue or carbonized fibrous tissue, which were considered pathologically negative. The rates of pathological positivity for the two groups were 91.67% (33/36) and 87.10% (27/31), respectively, with no statistically significant difference (p=0.6955). The rates of positive malignancy were 72.22% (26/36) and 77.42% (24/31) of two groups, respectively, with no statistically significant difference (p=0.2276). At 6-, 18-, and 24-months post-procedure, the local control rates for group A and group B were 100.0% vs. 100.0%, 94.44% (34/36) vs. 96.77% (30/31), and 86.11% (31/36) vs. 87.10% (27/31), respectively, with no significant differences (p=0.2156).

**Table 3:** Details of treatment efficacy.

Characteristics	Group A (n = 36)	Group B (n = 31)	P-value
Technique efficacy rate	36(100)	31(100)	>0.9999
Positive biopsy rate	33(91.67)	27(87.10)	0.6955
Complete ablative rate, n (%)			0.2156
1m	36(100)	31(100)	
3m	36(100)	31(100)	
6m	36(100)	31(100)	
12m	36(100)	31(100)	
18m	34(94.44)	30(96.77)	
24m	31(86.11)	27(87.10)	

### Bleeding and thrombosis-related complications

**Figure 2:**
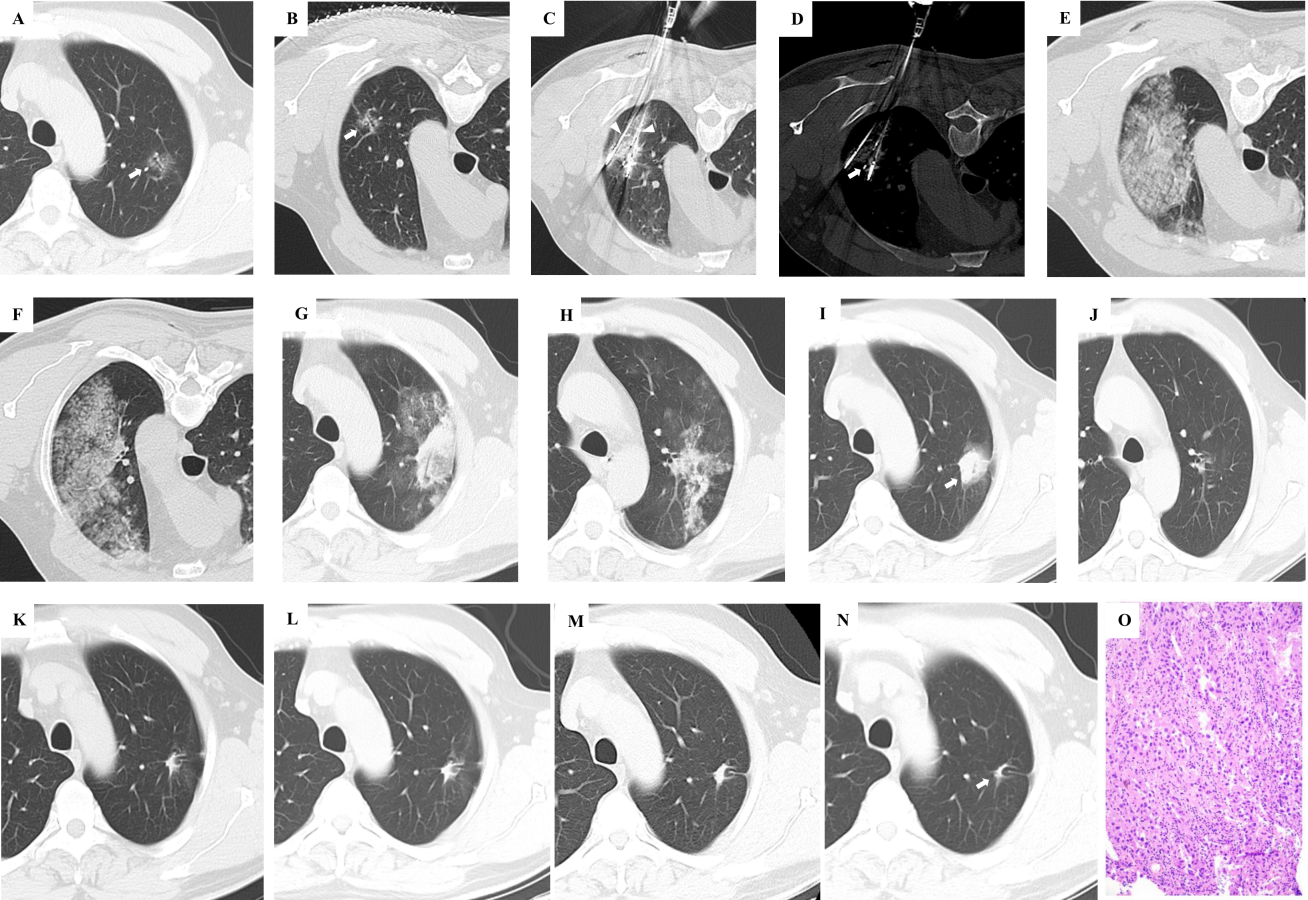
This 67-year-old male patient, with a history of long-term oral aspirin use post-percutaneous coronary intervention, presented with a highly suspicious malignant GGO in the left lung lobe. Upon expressing a clear preference against surgical resection, CT-guided synchronous percutaneous biopsy and microwave ablation (MWA) were chosen as an alternative therapeutic approach. **(A)** A GGO with a maximum diameter of 18.1mm is identified in the left upper lung lobe (indicated by the white arrow). **(B)** For the procedure, the patient was positioned prone. Imaging was aligned, and a puncture plan was meticulously outlined. **(C)** Two antennas were strategically placed at the predetermined locations as per the preprocedural plan. **(D)** The biopsy needle was accurately positioned at the designated site in accordance with the preprocedural plan. **(E)** Following the removal of the antenna and biopsy needle, a significant area of pulmonary parenchymal hemorrhage was noted. **(F)** Extensive hemorrhage within the lung parenchyma was observed at various CT scan levels. **(G)** A 24-hour follow-up CT scan revealed that the majority of the parenchymal hemorrhage had been resorbed. **(H)** A 24-hour CT scan at different levels also showed substantial absorption of the parenchymal hemorrhage. **(I)** One-month post-procedure, a CT scan indicated that the pulmonary parenchymal hemorrhage had been entirely resolved. The ablation zone completely enveloped the original GGO, deemed a successful ablation. **(J)** Parenchymal hemorrhage at multiple CT levels had also been fully resorbed. **(K)** At three months, a CT scan demonstrated that the ablation area had commenced shrinkage, with no residual or recurrent GGO detected. **(L)** A six-month CT scan revealed ongoing shrinkage of the ablation area. **(M)** The twelve-month CT scan indicated continued reduction in the size of the ablation area. **(N)** By eighteen months, the ablation area had transformed into a scar-like lesion, signifying the stability of the treatment outcome. **(O)** The pathology of this GGO was invasive adenocarcinoma.

**Table 4** shows the details of bleeding and thrombosis complications. The overall incidence of major complications was 9/36 (25.00%) in group A and 12/31 (38.71%) in group B. In group B, there were two cases of grade 4 thrombotic complications—one case of cerebral infarction and one case of pulmonary embolism. The two patients recovered after adequate anticoagulant treatment and hospitalization observation, without the need for interventional embolization or surgical intervention. No grade 5 complications occurred. The incidence and severity of major complications did not differ significantly between the two groups. Major bleeding complications were observed in 15 patients (8 in group A and 7 in group B), and major thrombotic complications occurred in 6 patients (1 in group A and 5 in group B), with no significant difference in incidence. Statistically significant differences were found in International normalized ratio (INR) and D-dimer levels between the two groups on the 3rd day post-procedure, as well as INR on the 30th day post-procedure. The maximum bleeding area in group A was slightly larger than that in group B both during the procedure and 24 hours post- procedure, although the difference was not statistically significant. **Figure 2** presents the serial imaging findings and corresponding pathological specimens from a 67-year-old male patient with long-term aspirin use, demonstrating the perioperative and postoperative course following synchronous biopsy and MWA of a highly suspicious malignant GGO in the left lung. **Table 5** presents an analysis of perioperative bleeding and thrombotic complication risks based on different preoperative anticoagulation regimens. When anticoagulation strategy was analyzed as a variable, no statistically significant differences were observed in perioperative bleeding or thrombotic complication rates between the two groups.

**Table 4:** Details of bleeding and thrombotic complications.

Characteristics	Group A (n=36)	Group B (n=31)	P value
Total major bleeding and thrombotic complications*, n (%)	9 (25.00)	12 (38.71)	0.4265
Grade of major complications			0.2697
Grade 3	9 (25.00)	10 (32.26)	
Grade 4	0	2 (6.45)	
Grade 5	0	0	
Periprocedural major bleeding complications (5 days before to 3 days after procedure), n (%)	7 (19.44)	5 (16.13)	0.7609
Delayed major bleeding complications (3 days after procedure), n (%)	1 (2.78)	2 (6.45)	0.5704
Days (after procedure) to delayed major bleeding complications,days	3.63 ± 1.79	4.17 ± 2.75	0.6874
Total major thrombotic complications, n (%)	1 (2.78)	5 (16.13)	0.0882
Periprocedural major thrombotic complications, n (%)	0	1 (3.23)	0.5577
Delayed major thrombotic complications, n (%)	1 (2.78)	4 (12.90)	0.5903
Days (after procedure) to delayed major thrombotic complications, days	12.64 ± 6.22	9.63 ± 5.73	0.5875
Coagulation function at 3 days after procedure			
Prothrombin time (s)	10.87 ± 0.65	10.79 ± 0.47	0.5761
Activated partial thromboplatin time (s)	38.29 ± 5.66	39.08 ± 4.32	0.7548
International normalized ratio	1.07 ± 0.34	0.92 ± 0.04	0.0273
D-dimer (μg/L)	0.37 ± 0.30	2.62 ± 0.75	0.0157
Coagulation function at 30 days after procedure			
Prothrombin time (s)	11.04 ± 0.75	11.01 ± 0.75	0.8747
Activated partial thromboplatin time (s)	39.73 ± 4.88	37.22 ± 4.67	0.2836
International normalized ratio	1.14 ± 0.38	0.94 ± 0.07	0.0078
D-dimer (μg/L)	0.35 ± 0.24	0.34 ± 0.21	0.7933
Characteristics of hemorrhagic area, cm^2^			
Mean MAX hemorrhagic area at procedure	12.55 ± 7.92	10.25 ± 4.40	0.1556
Mean MAX hemorrhagic area 24 hours after procedure	8.46 ± 5.30	6.45 ± 2.88	0.0637
Absorbed hemorrhagic area 24 hours after procedure	4.09 ± 4.59	3.80 ± 3.41	0.7743
Hemorrhagic area increased 24 hours after procedure, n (%)	4 (11.11)	2 (6.45)	0.6784

Quantitative variables are expressed as means ± standard deviation.

*Major complications were defined as those of grade 3-5 according to the Cardiovascular and Interventional Radiological Society of Europe (CIRSE).

**Table 5:** Details of periprocedural complications across different anticoagulant regimens.

Anticoagulant Regimen (%)	Group A (n=36)	Group B (n=31)	Bleeding complications	Thrombotic complications	P value
Monotherapy	30 (83.3)	26 (83.9)			0.1324
Aspirin	12 (33.3)	11 (35.5)	4	1	
Clopidogrel	8 (22.2)	6 (19.4)	3	1	
Warfarin	4 (11.1)	3 (9.7)	2	1	
Rivaroxaban	3 (8.3)	3 (9.7)	1	1	
Apixaban	2 (5.6)	2 (6.5)	1	0	
Dabigatran	1 (2.8)	1 (3.2)	0	0	
Combination Therapy	6 (16.7)	5 (16.1)			0.1071
Aspirin + Clopidogrel	4 (11.1)	3 (9.7)	3	1	
Aspirin + Rivaroxaban	1 (2.8)	1 (3.2)	1	0	
Clopidogrel + Warfarin	1 (2.8)	1 (3.2)	1	1	
Total	36 (100)	31 (100)	14	7	

## Discussion

Image-guided thermal ablation is increasingly popular for the treatment of pulmonary GGOs. Current clinical evidence suggests that GGO-like lung cancer has a very low rate of lymph node and distant metastasis, making local lesion inactivation a viable treatment option (2, 26, 27). Furthermore, GGO-like lung cancer often as multiple GGOs, which would benefit from the ultra-minimally invasive, repeatable, and high local control rates associated with thermal ablation techniques (28, 29). Synchronous B+MWA is a commonly employed method for pulmonary GGOs (30–32). CT-guided puncture relies on the excellent visualization of GGOs on CT scans and precise puncture technique. However, the pulmonary parenchymal bleeding surrounding the GGOs caused by the puncture and biopsy can obscure the CT visualization of the nodules and potentially impacting treatment effectiveness (33, 34). Patients on antithrombotic therapy are at a higher risk of developing bleeding during puncture due to abnormal platelet function (35, 36). However, arbitrarily stopping antithrombotic medications can lead to an increased risk of thrombotic diseases (37, 38). These patients may develop several challenges: 1) Pulmonary parenchymal bleeding can impede the localization of GGOs; 2) The use of hemostatic agents following bleeding may further promote the development of thrombotic complications; 3) Persistent bleeding can result in extended hospital stays and other complications. Consequently, appropriate management of antithrombotic therapy during the perioperative period is crucial for patients with GGOs who are on antithrombotic therapy.

The present study enrolled patients with pulmonary GGOs receiving antithrombotic therapy who underwent B+MWA procedures. A case-control analysis revealed no significant baseline difference between Group A patients who received rivaroxaban as a bridging therapy and Group B patients who abruptly ceased antithrombotic therapy during the perioperative period. The technical success rate was 100%, with satisfactory rates of positive biopsy rate and local control rate. Perioperative complications showed a similar incidence of bleeding events in both groups, though the incidence of thrombotic events was higher in Group B than in Group A, a difference that didn’t reach statistical significance. The thrombotic events in Group B comprised two severe complications: one case of cerebral infarction and one case of pulmonary embolism, with mild sequelae following appropriate treatment. Previous study by Anderson R et al. has highlighted the high risk of thrombosis in lung cancer patients and the potential benefits of antiplatelet agents in cancer adjuvant therapy (39). A few studies have reported that patients with a history of thrombotic disease experienced a significantly increased risk of subsequent thrombotic complications after cessation of continuous antithrombotic therapy, and a strategy of replacing antithrombotic drugs with perioperative anticoagulants is recommended (40–42). The lower incidence of thrombotic complications in Group A patients may be attributed to the use of rivaroxaban as a bridging therapy during the interruption of antithrombotic therapy and the prompt resumption of antithrombotic therapy thereafter. Chen et al. reported in case-based narrative review that the decision to discontinue or maintain antithrombotic therapy should be informed by the individual patient’s risks of bleeding and thrombosis, as well as the specific procedural risks involved. For procedures with a low risk of bleeding, anticoagulant therapy can be safely maintained without disruption. Conversely, when anticoagulant therapy cannot be safely continued, careful consideration should be given to the timing of interruption and resumption of therapy, along with the potential need for bridging therapy to mitigate any increased risk of thrombosis (43). Similarly, many studies also support the use of anticoagulant bridging antithrombotic agents to manage the balance of bleeding and thrombotic complications in patients receiving antithrombotic therapy during the perioperative period (44–46). In summary, perioperative rivaroxaban bridging therapy for B+MWA in patients with pulmonary nodules on antithrombotic therapy effectively reduces the incidence of thrombotic complications while maintaining an acceptable rate of bleeding complications.

The present study has several limitations. Firstly, it was conducted at a single center, which means the findings may not be uniformly replicable in other clinical settings. Secondly, the study excluded patients with pulmonary GGOs measuring <8 mm and >30 mm, as well as those with multiple lesions treated using the same procedure. These exclusions could potentially explain the high technical success rate and low complication rate observed in our research. Thirdly, our sample size was limited by the inclusion of various antithrombotic drug classes, without accounting for the metabolic characteristics of each medicine. Additionally, the conventional coagulation tests presented in this study, including PT, APTT, INR, and D-dimer, are not sensitive indicators of rivaroxaban’s anticoagulant activity. It is therefore recommended that specific anti-factor Xa activity assays be considered essential monitoring tools for assessing the anticoagulant effects of rivaroxaban in future prospective studies. Furthermore, randomized studies with larger sample sizes are warranted, and standardized management protocols for rivaroxaban bridging therapy should be carefully redefined and optimized in subsequent research.

In conclusion, for patients with pulmonary GGOs who are on antithrombotic therapy, the use of oral rivaroxaban as a bridging strategy during perioperative period for simultaneous CT-guided percutaneous B+MWA is both safe and effective. This approach can lower the incidence of severe thrombotic complications during the perioperative period, compared to the abrupt discontinuation of antithrombotic therapy during this time.

## Data Availability

The datasets used and/or analyzed during the current study are available from the corresponding author on reasonable request.
